# Calorimetry in Computed Tomography Beams

**DOI:** 10.6028/jres.126.054

**Published:** 2022-03-10

**Authors:** H. Heather Chen-Mayer, Ronald E. Tosh, Fred B. Bateman, Paul M. Bergstrom, Brian E. Zimmerman

**Affiliations:** 1 National Institute of Standards and Technology, Gaithersburg, MD 20899, USA

**Keywords:** absorbed dose, calorimeter, CT dose, medium energy X-rays, polystyrene, thermistor

## Abstract

A portable calorimeter for direct realization of absorbed dose in medical computed tomography (CT) procedures was constructed and tested in a positron emission tomography (PET) CT scanner. The calorimeter consists of two small thermistors embedded in a polystyrene (PS) cylindrical “core” (1.5 cm diameter) that can be inserted into a cylindrical high-density polyethylene (HDPE) phantom (30 cm diameter). The cylindrical design of core and phantom allows coaxial alignment of the system with the scanner rotation axis, which is necessary to minimize variations in dose that would otherwise occur as the X-ray source is rotated during scanning operations. The core can be replaced by a cylindrical ionization chamber for comparing dose measurement results. Measurements using the core and a calibrated thimble ionization chamber were carried out in a beam of 6 MV X-rays from a clinical accelerator and in 120 kV X-rays from a CT scanner. Doses obtained from the calorimeter and chamber in the 6 MV beam exhibited good agreement over a range of dose rates from 0.8 Gy/min to 4 Gy/min, with negligible excess heat. For the CT beam, as anticipated for these X-ray energies, the calorimeter response was complicated by excess heat from device components. Analyses done in the frequency domain and time domain indicated that excess heat increased calorimetric temperature rise by a factor of about 15. The calorimeter’s response was dominated by dose to the thermistor, which contains high-atomic-number elements. Therefore, for future construction of calorimeters for CT beams, lower-atomic-number temperature sensors will be needed. These results serve as a guide for future alternative design of calorimeters toward a calorimetry absorbed dose standard for diagnostic CT.

## 1 Introduction

Standard reference dosimetry of radiotherapy beams using gamma rays or high-energy X-rays in the mega-electron volt (MeV) range is based on the quantity absorbed dose to water [1], which is realized with a water calorimeter as the primary standard [2]. This was not always true: Prior to the advent of calorimetry for radiation dosimetry, such primary standards were based on air kerma (kinetic energy released per unit mass). The move from air kerma– to absorbed dose–based standards for these radiotherapy applications was motivated by the desire to reduce the uncertainty of clinical reference dosimetry [3].

This contrasts with the situation for standard reference dosimetry of medical radiation using medium-energy X-rays (tube voltage 80 kV to 300 kV), which still relies on air-kerma standards [4, 5]. Much progress has been made by metrology institutes around the world in adapting water calorimetry for medium-energy X-ray beams, as summarized in a recent comprehensive review article [3] highlighting the need for direct measurement of absorbed dose in these energy ranges. However, the emphasis has been on dosimetry of radiotherapy beams, leaving adaptations needed for calorimetry of diagnostic imaging radiation over this energy range open for further research.

In this work, we explored the use of calorimetric methods for realizing absorbed dose in the medium-energy X-ray range for an important modality in diagnostic imaging—computed tomography (CT). Realizing absorbed dose in a CT scanner is challenging because of the geometry of dose delivery: CT X-rays are presented in a fan or cone beam that rotates around the patient. This differs markedly from the static, spatially uniform dose fields required by most reference-dosimetry protocols. In diagnostic procedures like CT, the dose may be less than 0.1% of that used in radiotherapy and in standard reference dosimetry. Although the range of CT doses is orders of magnitude lower than that for therapy doses, dose *rates* are within an order of magnitude of those commonly used in standard reference conditions for therapy-level radiation. Therefore, current calorimetric methods should be suitable for CT dosimetry provided they can be adapted for use in a rotationally scanned field.

Aside from its potential to provide a new absorbed dose standard for CT, calorimetry may be useful for reducing uncertainties affecting current CT dosimetry protocols. The computed tomography dose index (CTDI) served as the *de facto* standard as a measure of the machine output until its recent proposed replacement by the American Association of Physicists in Medicine (AAPM) TG-200 phantom, which was devised to address inadequacies in representing dose to patient [6, 7]. The new protocol has a more advanced phantom design to capture the timing characteristics of the spiral source movement as the phantom advances during a scan, as well as to better establish dose equilibrium by capturing the scatter tails in modern CT with wider beams. Nevertheless, these standards are all based on ionization chambers (ICs) calibrated for air kerma in a static beam. To better represent dose to patient, IC measurements in a phantom (as is the case with the TG-200 phantom) need to be corrected for radiation absorption and scattering characteristics of the host material, based on the CT spectrum and filtration. This is commonly done by Monte Carlo simulations, which indicate vastly different outcomes for different clinical systems and large variations of dose to phantom depending on the material and size [8]. Therefore, a direct measurement of the absorbed dose in a CT beam inside of the newer phantoms would be instructive.

The present study outlines a novel attempt to adapt calorimetry methods for use in medical CT imaging to quantify absorbed dose in a phantom. The geometry and material composition of the phantom and core detection element were designed for compatibility with currently accepted CT dosimetry protocols (in commercial CT scanners) to evaluate the prospects for further development as an absorbed dose standard. Since this work involved adapting calorimetry methods used successfully for radiotherapy beams, testing was done both in radiotherapy and CT beams over a range of dose rates. For the former, the interpretation of results was straightforward, and good agreement was obtained between the calorimeter and the IC measurements. For the CT measurements, excess heat in the thermistors, due to enhanced absorption and heat production of the thermistor material (relative to the polystyrene [PS] core) induced by the lower-energy X-rays, introduced artifacts that obscured the desired signal of dose to the PS core. Both time-domain and frequency-domain methods were used on measured data to estimate the magnitude of artifacts and signal for purposes of comparing calorimeter measurements with chamber measurements. We present these results along with several indicators for future improvement in device design from analytical and numerical modeling.

## 2 Calorimeter Design and Measurement Methods

Compared to water calorimetry, a solid core calorimeter is more rugged and portable, has fewer complications in measurement setup, and presents no spill hazards for electrical components. Polystyrene was chosen because of its low heat defect associated with radiation-induced chemical reactions wherein heat contributions obscure the desired signal from energy imparted by radiation to the target. A hybrid PS-water calorimeter was previously constructed with PS disks submerged in water [9]. Constructed using the same stock PS as in Ref. [9], the current calorimeter core had a 1.46 cm diameter by 2 cm long cylinder with two 0.3 mm diameter thermistors (nominal composition Cu_0.8_Mn_2.16_O_4_) embedded inside the cylinder. The core was threaded onto a PS rod with a central channel through which electrical wires could be threaded. The assembled rod was inserted into a small channel of a section of the polyethylene (PE) cylindrical phantom, along its central axis for in-beam measurement. Therefore, while this could be considered a hybrid PS-PE calorimeter, we refer it as a PS calorimeter because the temperature rise detected by the embedded thermistors should be largely determined by the radiation response of the core material. The PS core can also be submerged in water, described below in Sec. 2.1.

**Fig. 1:**
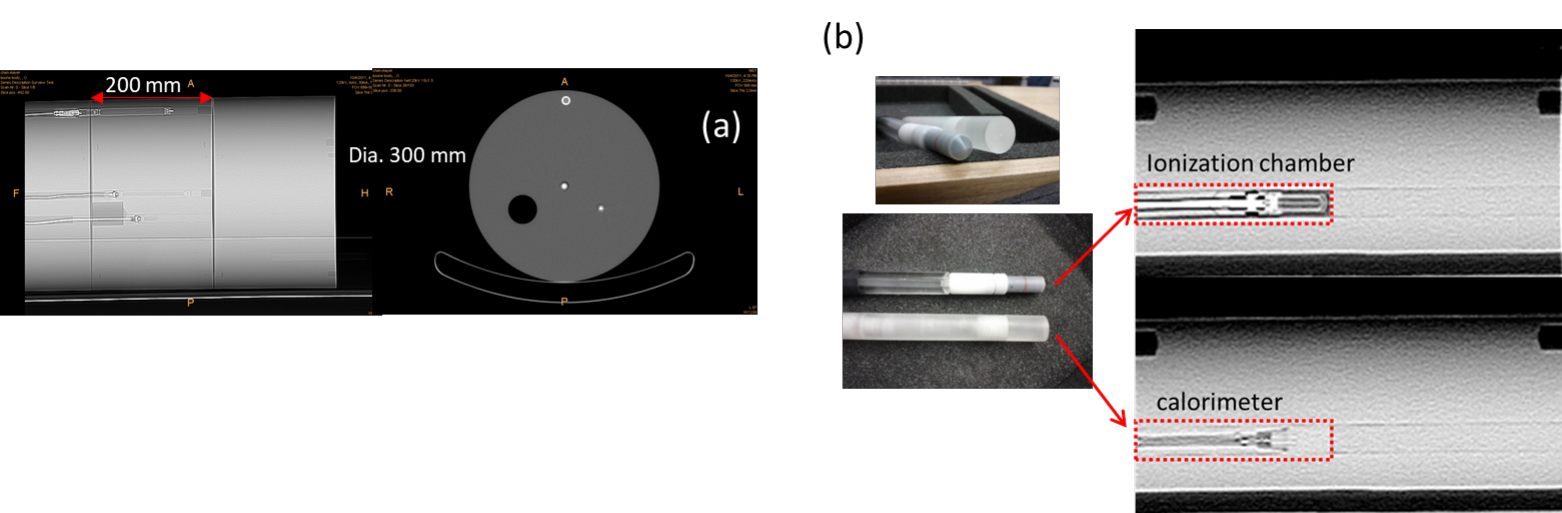
(a) CT image of the three-section high-density polyethylene (HDPE) phantom designed for dose measurement at various insertion points in the body. (b) Photographs of the polystyrene calorimeter probe with two embedded thermistors and an ionization chamber, and their respective CT images. The chamber and calorimeter probes can be swapped into the phantom for measurements sequentially or placed opposite each other in the channel for simultaneous measurement.

The phantom used in this work is an earlier version of the three-section (30 cm diameter, thicknesses of 10 cm, 20 cm, and 20 cm, respectively), high-density polyethylene (HDPE) phantom specified in Ref. [7] (where the final design consisted of three 20 cm thick sections) (Fig. 1). For measurement convenience, the 10 cm and the 20 cm thick sections were used separately and interchangeably in this work, because small differences in dose from longitudinal scattering (parallel to the axis of the phantom) within these two sections can be neglected for the purposes of this investigation.

**Fig. 2:**
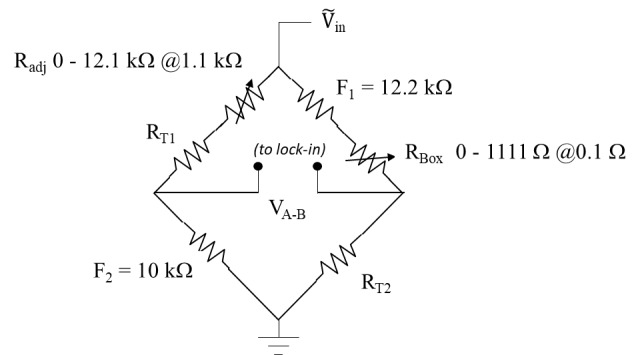
Calorimetry measurement schematics: A micro-Kelvin temperature rise can be measured with a Wheatstone bridge in conjunction with a lock-in amplifier. The temperature change is converted to radiation dose using the heat capacity of the calorimeter. The bridge balance and data acquisition were controlled by a LabVIEW program. The symbols F_1_ and F_2_ are fixed resistors; R_adj_ and R_Box_ are coarse and fine adjustable resistor banks for bridge balance; R_T1_ and R_T2_ are the temperature-sensing thermistors.

The measurement of small temperature changes is based on an AC-powered Wheatstone bridge with lock-in detection developed for the water calorimeter. As shown in Fig. 2, the two thermistors for temperature sensing are wired into opposite arms of the Wheatstone bridge. Phase-sensitive lock-in detection enables temperature sensitivity in the micro-Kelvin range at room temperature. The temperature rise in the PS is measured by converting the thermistor resistance change into temperature using temperature coefficients derived from a separate calibration procedure using a high-precision water bath. To obtain the absorbed dose to PS, the temperature rise for each cycle is multiplied by the heat capacity of PS at that instantaneous temperature, *Cp*(*T*), in J/kg-K, based on the following empirical relationship from Ref. [9]:

$Cp(T)\;=1000/M\;(a\;T^{-2}+bT+c)$, (1)

where *T* is the absolute temperature. With *M* = 104.15 g/mol (the molar mass of PS), *a* = 7.755 × 10^5^, *b* = 0.5345, and *c* = −41.58, *Cp*(*T*) is given in units of J/kg-K. *Cp*(*T*) is calculated at each cycle for the instantaneous temperature, *e.g.*, *Cp*(*T*) = 1212.3 J/kg-K at 24.5 °C.

While the main objective was to develop a calorimeter for CT beams used for medical imaging, we also tested this PS calorimeter in a 6 MV photon beam modality commonly used for cancer therapy, for which the operation of the National Institute of Standards and Technology (NIST) water calorimetry standard is well established [10−12]. Comparison measurements were made using the PS calorimeter and a calibrated IC. For the 6 MV beam, this was an Exradin A12 Farmer-type chamber^1^1 Certain trade names and commercial products are identified in this paper to specify the experimental procedures in adequate detail. This identification does not imply recommendation or endorsement by the authors or by the National Institute of Standards and Technology, nor does it imply that the products identified are necessarily the best available for the purpose. calibrated in terms of absorbed dose to water in a ^60^Co gamma-ray beam and converted later to dose to water in a 6 MV photon beam *via* the formalism specified in AAPM TG-51 [1]. A further conversion from dose to water to dose to HDPE in a 6 MV photon beam was needed to obtain dose to the HDPE phantom (from measurements obtained with the A12 IC inserted into the HDPE phantom). This was done using Monte Carlo simulations of 6 MV photon irradiation of the HDPE phantom, assuming the standard operation parameters of 10 cm by 10 cm field based on a full simulation of the electron accelerator and the treatment head. The energy absorbed per incident photon was calculated for the same small volume (1.5 cm diameter by 1 cm long, comparable to the volume of interest) inside of a 30 cm diameter by 20 cm HDPE phantom. The dose (energy per unit mass) to the small volume was calculated for PS (1050 kg/m^3^) and for water (1000 kg/m^3^), yielding a ratio (PS/water) of 0.962 (0.08%), which is in line with the previous measured ratio of 0.97 (1.2%) using a PS-water calorimeter [9].

For the 120 kV CT beam, calorimeter measurements were compared to dose measurements obtained from a Radcal RC0.6CT thimble IC that had been calibrated to air kerma in NIST M80−M150 X-ray beam qualities.

### 2.1 Measurements in 6 MV Photon Beam

**Fig. 3:**
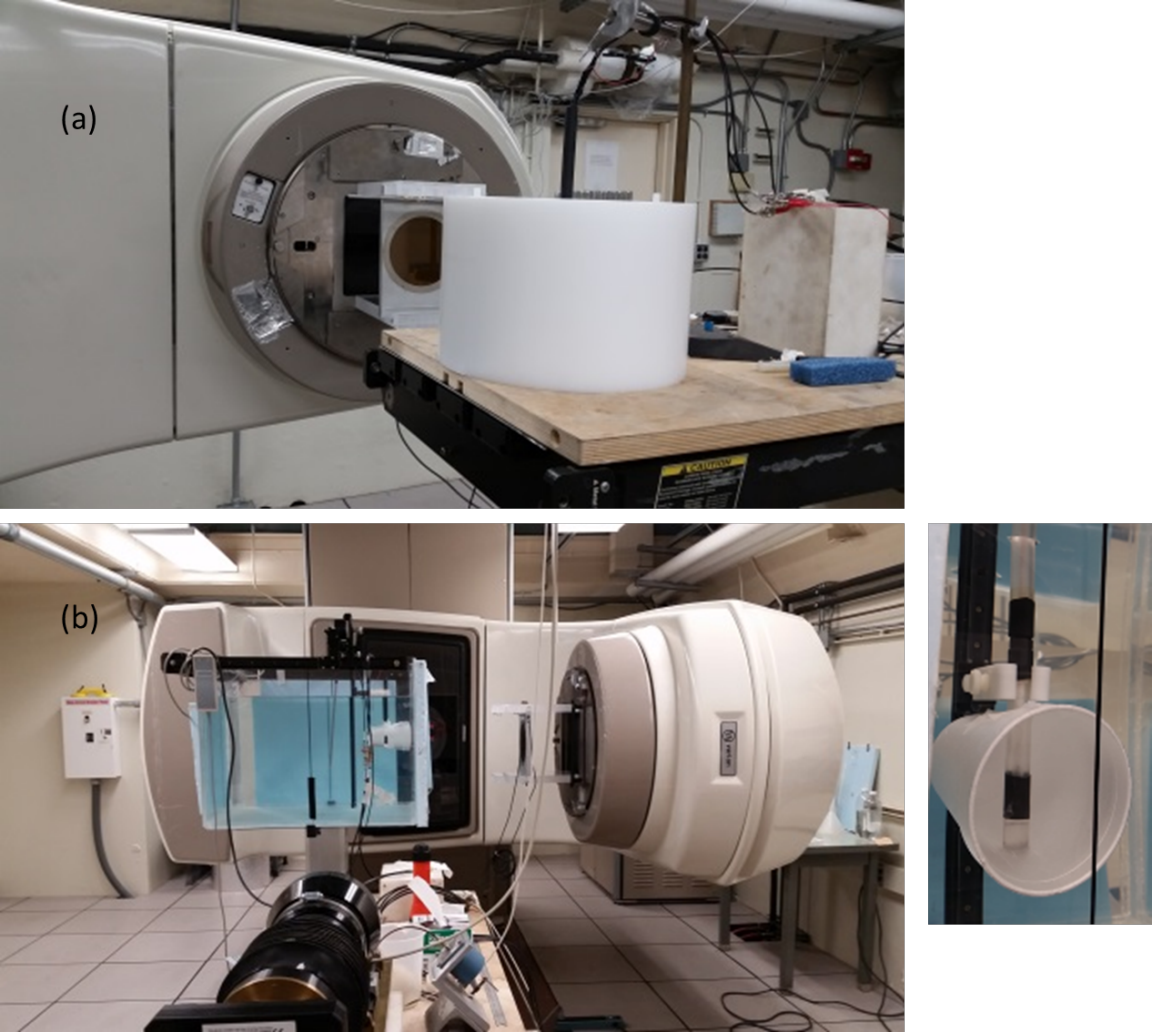
Photographs of the measurement in the high-energy photon beam from a clinical therapy machine. The photon field size is 10 cm by 10 cm, incident horizontally. (a) Setup for the PE phantom, which has a channel along the central axis allowing insertion of the ionization chamber or the calorimeter core. (b) In-water phantom measurement, where the calorimeter is inserted vertically in the water, adjusting the distance to 10 cm water-equivalent thickness. The close-up photo, on the lower right, taken through the wall of the water tank, shows the submerged calorimeter core surrounded by a Styrofoam cup as a convection barrier.

While the goal of this work was to develop calorimetry for the CT beam, we first tested the calorimetry assembly in a more practiced modality using high-energy photons in a uniform, static field to verify that the calorimeter was working as intended. Measurements were conducted at the NIST medical clinical linear accelerator (Varian 2100 C), or Clinac, in a 10 cm by 10 cm photon field, incident on the phantom horizontally (Fig. 3). The probes were inserted into the phantom along the cylindrical axis. The Clinac was programmed to deliver a range of dose rates, from 0.8 Gy/min to 4 Gy/min, to assess the linearity of the calorimeter response.

For the first set of measurements, the PS calorimeter was placed inside a section of the HDPE phantom [Fig. 3(a)]. Calorimetry measurements were performed at five nominal dose rates (0.8, 1.6, 2.4, 3.2, and 4.0 Gy/min, set by the Clinac’s monitor units), and for each dose rate, a sequence of pulses was delivered with a 60 s on/60 s off timing. There were 10 radiation cycles at each dose rate, except for the 2.4 Gy/min, which had 20 cycles. The IC measurement was carried out in the same setting for comparison.

**Fig. 4:**
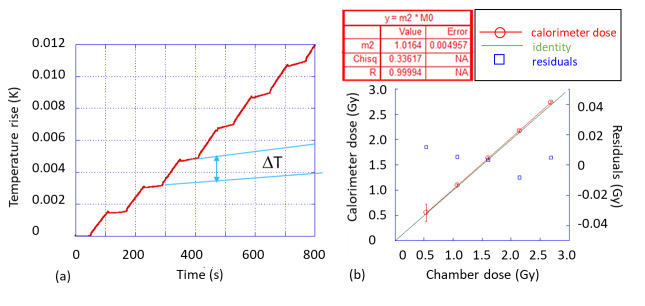
Measurements in 6 MV photon beam in polyethylene phantom. (a) Temperature-time waveform obtained from the calorimeter for multiple 60 s on/off cycles. (b) Calorimeter dose *vs*. IC dose at five dose rates, with a linear fit and residuals shown. The IC measured doses for the five nominal dose rates were 0.536 Gy, 1.07 Gy, 1.61 Gy, 2.15 Gy, and 2.69 Gy, respectively. The calorimeter dose value was the mean value of the 20 repeated cycles, and the uncertainty bars represent the standard deviation of the distribution over the 20 cycles.

Results for the HDPE phantom measurement are shown in Fig. 4. Figure 4(a) shows the measured temperature rise as a function of time for the first six cycles of the 20 cycles at one of the five dose rates, 2.4 Gy/min. The label Δ*T* is an indication of the temperature increase per cycle obtained using the standard midpoint extrapolation method [2]. The average temperature rise from these cycles was multiplied by the specific heat capacity of polystyrene to arrive at the absorbed dose in J/kg, or Gy. This was repeated for all five dose rates. The calorimeter dose thus measured is plotted against the corresponding IC measurements in Fig. 4(b), which shows a good linear response between the calorimeter dose and the IC measured dose, and a slope with a 1.6% (±0.5%) deviation from unity. As stated earlier, based on Monte Carlo simulation, the expected deviation from unity is about −3.8%. Taken together, these values indicate that there is an approximate bias of 5.5% prior to correction for systematic uncertainties, for example, heat transfer involving the PS core.

The second set of measurements was conducted in a water phantom, using the more familiar absorbed dose to water measurement protocol (TG-51). The photon field was incident on the water tank horizontally through the Plexiglas wall [Fig. 3(b)]. The PS calorimeter probe was positioned at 10 cm water-equivalent depth, accounting for the wall thickness. It was determined that at the higher dose rates (above 2.4 Gy/min), 60 s irradiation triggered natural convection in the water that produced spurious, slow temperature fluctuations in the probe. This was overcome by a combination of an improvised convection barrier [Fig. 3(b), right inset] and implementation of shortened irradiation cycles (from 60 s to 20 s on/off) for all dose rates. The IC measurements were made accordingly. Three sets of measurements were taken on three separate days. The number of cycles at each dose rate was 20 on day 1, when both thermistors were in use (full bridge), and 40 on days 2 and 3, when one thermistor became defective and was replaced by a fixed resistor (half bridge). It should be noted that the temperature changes determined are independent of the bridge settings, and indeed the results were in good agreement.

**Fig. 5:**
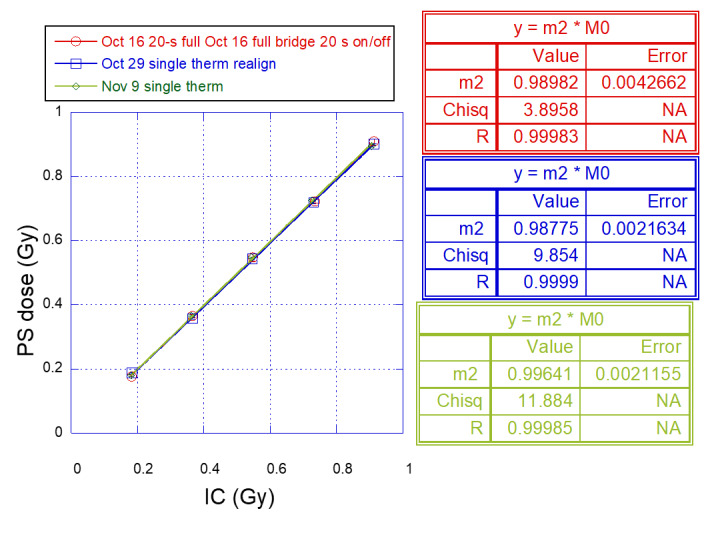
Measurements of dose in polystyrene (PS) calorimeter *vs*. ionization chamber of 6 MV photon beam in the standard water phantom, with an overlay of data from runs on three separate days (differentiated by color), showing excellent measurement reproducibility. The PS dose value was the mean value of the 20 (or 40) repeated cycles, and the uncertainty bars represent the standard deviation of the distribution over the 20 (or 40) cycles.

Three measurements using the PS calorimeter were made on separate days, along with the corresponding IC measurements, for which excellent repeatability was observed (Fig. 5). The slope (parameter “m2”) from each measurement deviated from unity by less than 1.2% (±0.4%).

### 2.2 Measurements in CT Beam

**Fig. 6:**
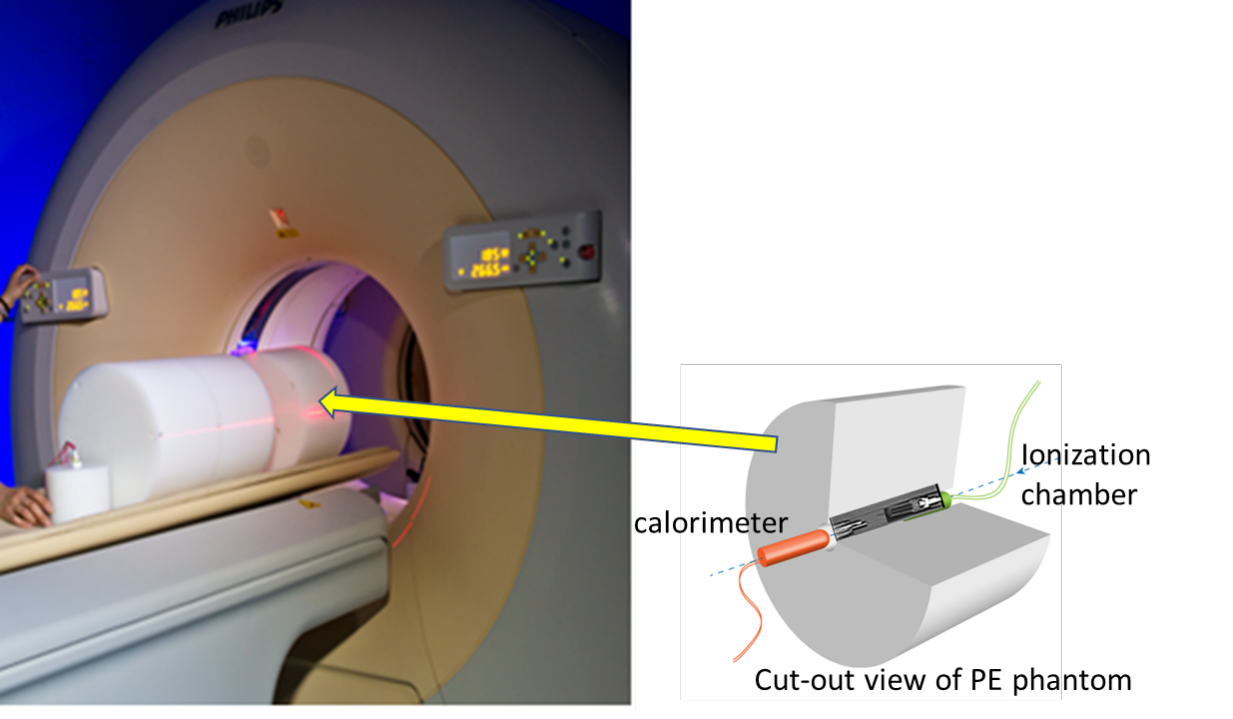
Photograph of a medical PET/CT scanner with the polyethylene phantom sections on the patient bed and an illustration of the phantom with the dose measurement devices inserted axially.

The CT measurements were performed in the CT portion of a Philips Gemini-TF PET/CT scanner with 16 slice CT. Figure 6 shows a photograph of the setup in the scanner and a drawing of the phantom measurement geometry.

The CT scanner operating parameters were 120 kV tube voltage, 350 mA current, and 16 mm by 1.5 mm collimation. The experimental run consisted of ten 2 s axial scans (one source rotating per “scan”), yielding a dose at the center of the HDPE phantom of about 8.6 mGy per scan. Note that, clinically, the current setting is usually below 150 mA, at the lower end of these measurements. We elevated the current to generate a higher signal-to-noise ratio for the calorimeter measurement, since the CT dose is much lower compared to the range of doses commonly measured using a calorimeter.

**Fig. 7:**
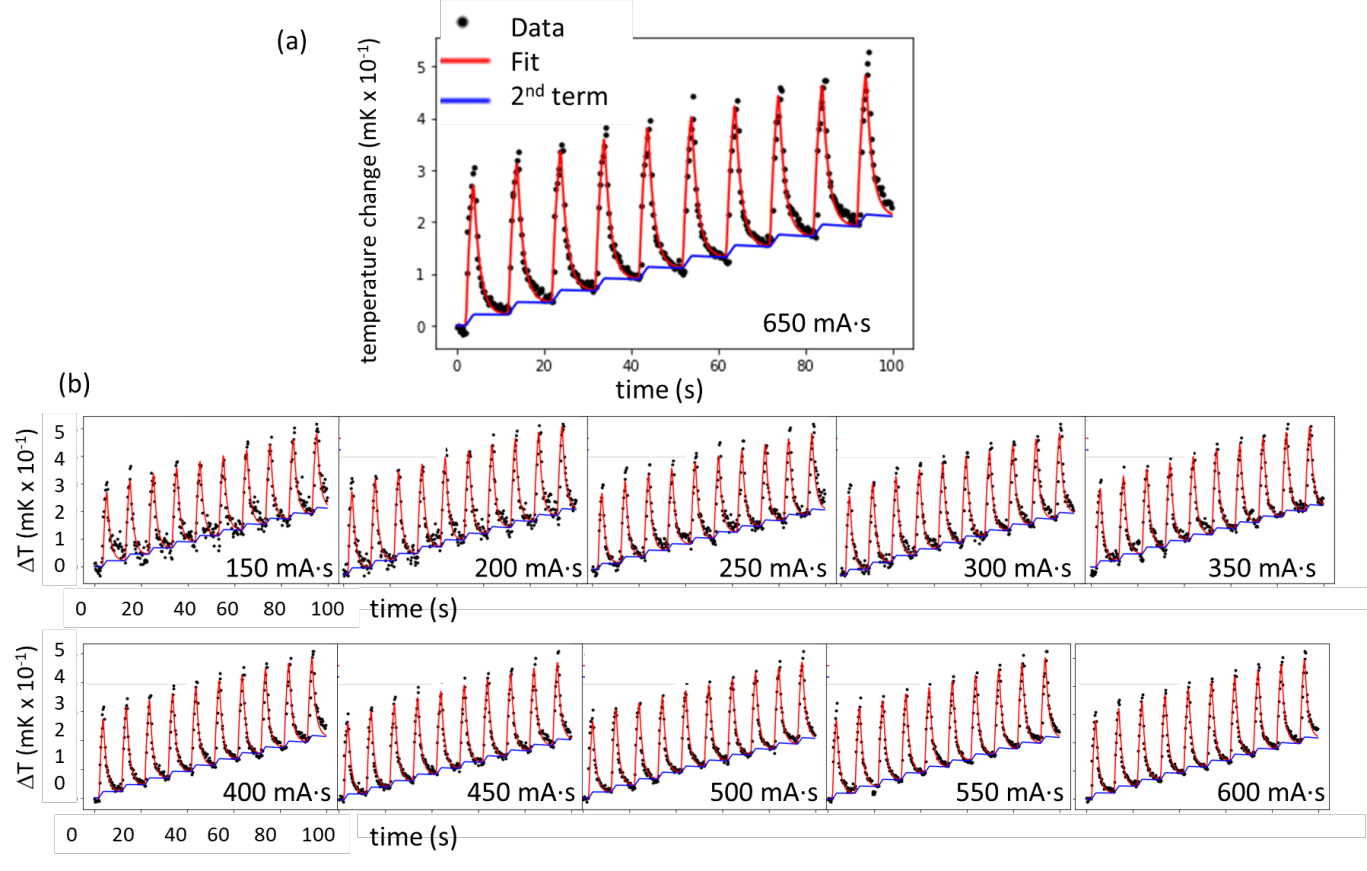
(a) Calorimetric response measured (dots) in the CT imaging system for the 650 mA·s dose-rate settings, along with the fit (solid line) using Eq. (2), which contains two terms: the fast (thermistor) and slow (PS) components. The lower staircase-like response is the second term, from which the temperature rise in the PS was determined. (b) The temperature waveforms for the rest of the entire series, all normalized to 100 mA·s, are also shown in the panels at the bottom.

Figure 7 shows the temperature waveforms obtained from the calorimeter in the CT at 11 different CT tube current settings (expressed as the product of X-ray tube current in mA and the exposure time in s), from 150 mA⋅s to 650 mA⋅s at 50 mA⋅s increments. All waveforms shown were normalized to the current-time product of 100 mA⋅s. The shape of the temperature response for each cycle is dominated by a large, transient peak due to radiation heating of the thermistor material and subsequent cooling due to conduction of heat from the thermistor to the surrounding PS core material. This obscures a much smaller underlying temperature step attributable to radiation heating of the PS core material. Because the heating and cooling segments in the waveforms are highly nonlinear, midpoint extrapolation could not be used to derive the per-cycle temperature increments (as was done with the 6 MV waveforms, shown in Fig. 4). We used two methods to analyze the data and to extract the dosimetry information.

In the first method, we attempted to account for the transient and step components evident in the time waveforms by convolving a function representing source strength and timing with a transfer function representing the impulse response of the thermistor to heat conduction to the PS core. For the latter, a simple, Newton’s law approach for heat conduction yielded separate exponential terms for conduction between the thermistor and core and between the core and the phantom. Accordingl*y*, the following semi-empirical functional form was used to fit the temperature waveform u(t):

$u(t)=(k_{1}\;e^{-t/\tau_{1}}{+\;k_{2}e}^{-t/\tau_{2}})*f(t)$, (2)

where the coefficients $k_{1}\;and\;k_{2}\;$(in units of mK/s) determine the relative weight of the two exponential terms of the calorimeter response, for which the time constants (*i.e.*, rise or decay times) $\tau_{1}$and $\tau_{2}$ are characteristic of the transient and step components, respectively. The unitless timing function *f(t)* is a unit-height square wave with zero baseline and a 20% duty cycle (which, in this work, was 2 s on and 8 s off). This function was used to fit the data along with a linear trend to account for the background drift for individual runs. The first term showed a rapid rise and decay, and the second term exhibited a staircase response, as shown in the lower blue curve in Fig. 7(a). Parameters $k_{1}\;and\;k_{2}$ were chosen such that a minimum chi-square was reached between the calculation and the experimental data. For each dose rate, the slope and offset of an underlying linear trend were used as fitting parameters to account for the temperature drift prior to onset of the radiation pulse sequence. Reasonable fits were obtained with the same $k_{1}\;and\;k_{2}$ by varying the slope and offset for data from each dose rate [Fig. 7(b)], suggesting the response was dose-rate independent. The fitted curves are shown as solid lines in Fig. 7. The fitted parameters are shown in Table 1. The ratio *k*_1_*/k*_2_ = 17 is commensurate with the ratio of transient-to-step signal components evident in Fig. 7. (The numerical function employed time steps of 0.25 s to match the experimental time step, which resulted in an 18% underestimate of the convolution integral, which was compensated by raising *k*_1_ and *k*_2_ by the same amount. However, this did not affect the ratio of *k*_1_*/k*_2_.) Additional interpretation of these fitted parameters is given in the Discussion (Sec. 3).

**Table 1:** Parameters determined from least-squares fit to the data in Fig. 7 using Eq. (2) with the 650 mA·s data set. Uncertainties for each parameter were determined by identifying parameter deviations such that the chi-square was doubled from the minimum.

Parameter	$\tau_{1}\;$(s)	$\tau_{2}\;$(s)	$k_{1}$ *(mK/s)*	$k_{2}$ *(mK/s)*
Value	1.4	628	0.27	0.016
Est. uncertainty	(−0.7, +0.6)	(−50, +86)	(−0.15, +0.06)	(−0.0017, +0.0013)

**Fig. 8:**
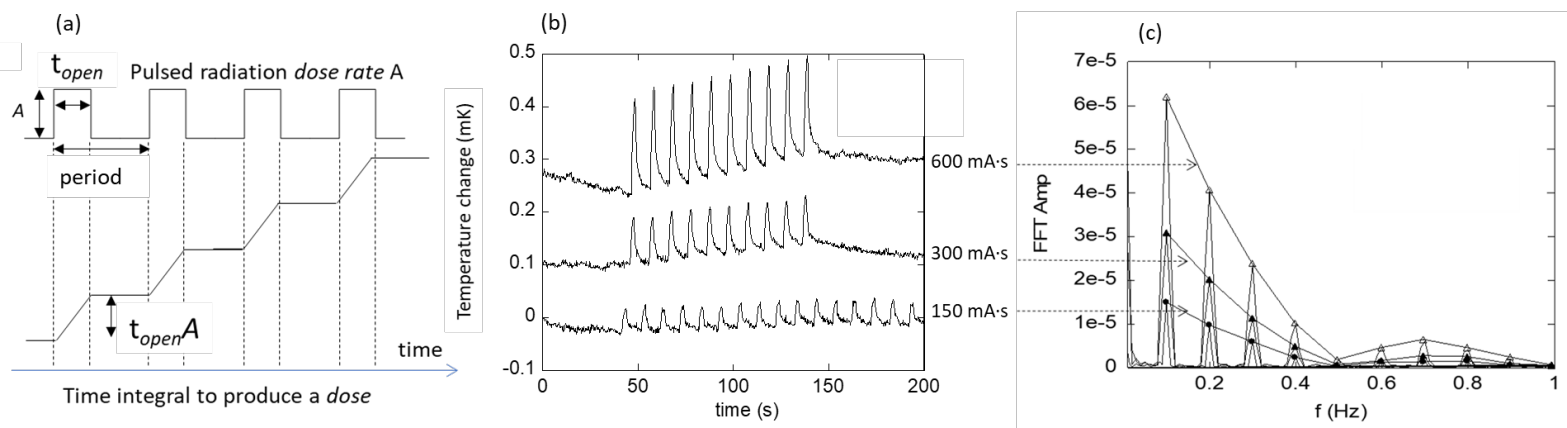
(a) Pulsed radiation modeled as a train of square waves, with the amplitude *A* proportional to the dose rate. Here, *t*_open_ is the beam-on time. The calorimeter response as a temperature waveform is the integral of that square wave, which in the absence of excess heat or heat transfer is a sloping staircase with an increase in height of *t*_open_⋅*Α* per cycle. (b) Temperature waveform obtained by the calorimeter at three CT current settings, a subset from Fig. 7. (c) Discrete Fourier transform of (b), wherein the signal is separated into frequency bins in the form resembling a modified *sinc* function. The relationship between the amplitudes in the two domains is shown in Eq. (3).

The second method takes advantage of the cyclic nature of the data by using a fast Fourier transform (FFT) into the frequency domain to quantify the total response in a model-independent way [10−12], as illustrated in Fig. 8. The per-cycle radiation-induced temperature rise is related to the fundamental (*n* = 1) FFT amplitude by

**Fig. 9:**
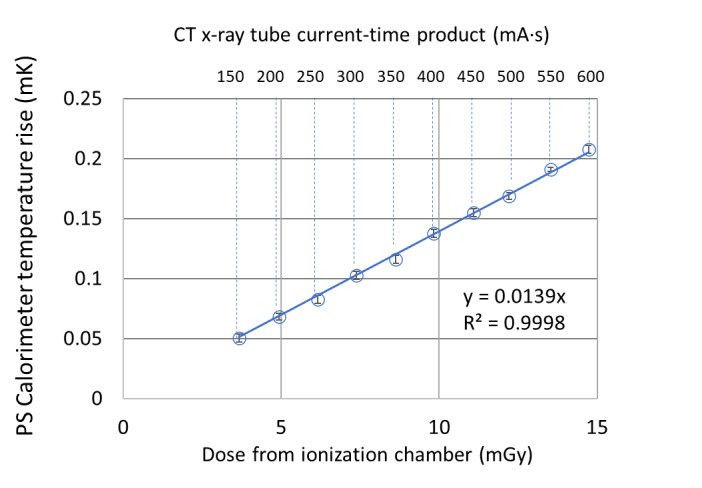
Total calorimeter temperature rise obtained from the FFT analysis (Fig. 8) *vs.* the IC reported dose. The error bars represent one standard deviation.

$FFT\;amp=\;A\;\frac{t_{open}}{n\pi }sinc\left(n\pi \frac{t_{open}}{period}\right)$, (3)

where *t_open_* is the beam-on time per cycle (2 s), *period* is cycle period (10 s), and A again represents the rate of temperature change during irradiation. [In principle, the right-hand side of Eq. (3) should include a factor representing the thermal transfer function of the calorimeter, but since we evaluated “FFT amp” at a single frequency, it would be a multiplicative constant and thus is absorbed into *A*.] The latter is plotted against the dose readout from the IC in Fig. 9, showing a good linear correlation. The corresponding current setting is also shown as the second *x* axis on top of the plot. The slope of that curve is 0.0139 mK/mGy-IC. Assuming a specific heat capacity of 1208 J/kg-K for PS, the expected value for the slope is 0.00083 mK/mGy. Therefore, the observed temperature increase is 16.7 times higher than that expected from PS alone. This is not entirely surprising given the more highly absorbing thermistor and wire materials in this energy region. This observed magnitude is in line with a previous investigation of thermistor materials in kilovolt X-rays, in which the energy absorption by the thermistor relative to water was estimated by Monte Carlo methods to be about 20 [13]. (A calculation based on XCOM-derived [14] energy production, μ_en_/ρ, weighted by a typical 120 kV CT spectrum and with an attenuation length of 15 cm yielded a dose ratio for PS to water of 0.93, which suggests that the ratio of thermistor to PS should be 7% higher than that for thermistor to water, raising the factor of 20 to 21.4.)

## 3 Discussion

The typical dose and dose rates in the 6 MV high-energy therapy beam and in the 120 kV CT beam are compiled in Table 2 for comparison. This work demonstrated that it is possible to obtain a linear response by the calorimeter prototype in two energy regions that are separated by nearly two orders of magnitude. However, it is also worth noting the very different mechanisms that govern radiation interactions in these two energy ranges: Photoelectric absorption is more important at the lower energy range, whereas Compton scattering and pair production are more important at the higher range of energies.

**Table 2:** Nominal dose and dose rates for the typical irradiation times used in this work. The dose values are based on IC measurements at the same location as the calorimeter. For the 6 MV beam, the chamber used was calibrated to absorbed dose to water. For the 120 kV beam, it was calibrated to air kerma.

Modality	6 MV (Clinac)	120 kV (CT)
Irradiation time per cycle (s)	20	2
Midrange dose per cycle (Gy)	0.55	0.0086
Range of dose rate measured (Gy/min)	0.56–2.73	0.11–0.41

The dominance of photoelectric absorption at CT beam energies leads to large, material-dependent variations in absorbed dose (owing to the dependence of the interaction cross section on atomic number, *Z*), as noted in other works [5]. Accordingly, the dose delivered to various high-*Z* calorimeter components, such as thermistors, considerably exceeds the dose delivered to the PS core material, and the excess heat created thereby produces large artifacts in calorimetry signals. The indication from the frequency-domain analysis above is that such excess-heat artifacts are responsible for an estimated radiation-induced temperature rise that is a factor of 16.7 higher than that expected from known material properties. Extracting the component due to temperature rise in the PS core is not possible in the frequency domain without additional assumptions; however, the time constants $\tau_{1}$ = 1.4 s and $\tau_{2}$= 628 s obtained from the time-domain analysis above may be helpful. When combined with thermal diffusivity, such time constants can be used to estimate the spatial dimensions of components within the calorimeter that contribute to the measured signal. For example, using a thermal diffusivity *δ* ≈ 1.0 × 10^−7^ m^2^/s [15] and the larger time constant*τ* = 628 s, one obtains an effective spatial dimension for heat conduction *r* ≈ $\sqrt{\delta \tau }$ [16] of about 8 mm, which is comparable to the 7.3 mm radius of the PS core. Similarly, for the thermistor (assumed to be MnO_2_ with a comparable thermal diffusivity [17]), the time constant of 1.4 s yields *r* ≈ 0.3 mm, which approximates the 0.15 mm thermistor radius. These estimates suggest that heat transfer is not negligible for our geometry and that a larger size PS core may be needed.

Assuming that the fast and slow signal components are linked to the thermal response of the thermistor and the PS core, respectively, an estimate of dose to the core may be obtained by midpoint-extrapolation analysis of the PS response [blue curve in Fig. 7(a)]. The result is on average approximately 0.030 mK per cycle per 100 mA·s. This temperature change multiplied by 1208 J/kg-K (the heat capacity of PS) gives a dose of 36 mGy per cycle per 100 mA⋅s, which is a factor of 15 higher than the IC-measured dose of 2.46 mGy per cycle per 100 mA⋅s. In frequency-domain analysis of the same data, the apparent dose obtained is also a factor of 17 higher than the IC dose. The merit of these analyses can be further debated after a more rigorous analysis (beyond the scope of this paper) involving solution of the heat equation for the proper model, both analytically and numerically (finite-element simulation), which is reserved for a future study.

Based on this study, future development of the CT calorimeter probe should focus on eliminating or greatly reducing the quantity of materials used in its construction that possess higher *Z* values (compared to PS) and thus contribute excess heat. Smaller thermistors may be difficult to characterize or to fabricate into the probe; however, it may be possible to replace thermistors with smaller temperature-sensing devices such as thin-film–type thermistors or radiation-hard photonic sensors, if they can be made to be sufficiently sensitive [18, 19]. To the extent that excess-heat artifacts remain a problem in calorimeter signals, it will be necessary to devise digital filtering to extract the desired signal component pertaining to dose to the PS core material. The analysis detailed here presents a starting point for more sophisticated spectral analysis techniques and/or time-domain convolution involving more accurate physical models of heat conduction.

Another factor necessary for this work is a thorough specification of material properties pertaining to radiation interactions in the core volume and subsequent heat transfer. We have already mentioned the need for Monte Carlo studies to convert chamber measurements from air kerma to dose to PS (or dose to water) for 120 kV photons; however, such studies also are needed to model dose to the calorimeter core and sensors in CT-like radiation. The latter will provide the basis for heat-source terms used in finite-element studies to obtain correction factors for heat transfer within the core. Such work will build on our earlier finite-element modeling of a rotating CT beam irradiating a cylindrical phantom [20], in which a dominant thermal response from the thermistor over the polystyrene was observed, in qualitative agreement with the time waveform analysis in this work.

## 4 Conclusion

The present work described the first steps in a project to develop a dose-to-water standard for diagnostic CT, beginning with a solid phantom modeled on the AAPM TG-200 standard for CT dosimetry, which is based on air kerma. We adapted techniques developed for use in primary standard water calorimeters for radiotherapy beams, which are based on absorbed dose. Testing of the prototype in 6 MV photon beams yielded reproducible results and showed good agreement with ICs calibrated for absorbed dose to water. For a calorimeter with a polystyrene (PS) core in such high-energy therapy beams, heat defects are believed to be negligible, while excess heat (and subsequent heat transfer) produces measurable perturbations that are small and are easily corrected. However, the much lower-energy spectra that characterize diagnostic CT beams gave rise to much larger thermal gradients within the calorimeter body (particularly at junctions of dissimilar materials) owing to the very large differences in energy absorption at the lower end of the spectrum. The calorimeter and phantom were tested in a 120 kV CT beam at 11 different X-ray tube current settings (which resulted in 11 dose rates). We observed that such distortions in measurements of temperature within the calorimeter body dominated the temperature rise in the calorimeter body itself. The time waveform exhibited highly transient rise-and-decay behavior, preventing the use of conventional midpoint extrapolation analysis. Frequency-domain analysis was employed to obtain an overall temperature rise, which was shown to be linear for all dose rates when plotted against the dose measured by the IC. However, the apparent dose from this analysis was about 17 times higher than the IC-measured dose, signifying a large contribution from excess heat. These results lead to the conclusion that other, lower-*Z* temperature sensors will be needed for this application.

The measurement data and analysis method described in this work provide a foundation for assessing the performance of a portable polystyrene calorimeter. This information may be useful for developing a future primary dose standard based on calorimetry for CT beams.
